# Detection of Atrial Fibrillation Using a Ring-Type Wearable Device (CardioTracker) and Deep Learning Analysis of Photoplethysmography Signals: Prospective Observational Proof-of-Concept Study

**DOI:** 10.2196/16443

**Published:** 2020-05-21

**Authors:** Soonil Kwon, Joonki Hong, Eue-Keun Choi, Byunghwan Lee, Changhyun Baik, Euijae Lee, Eui-Rim Jeong, Bon-Kwon Koo, Seil Oh, Yung Yi

**Affiliations:** 1 Seoul National University Hospital Seoul Republic of Korea; 2 Department of Electrical Engineering Korea Advanced Institute of Science and Technology Daejeon Republic of Korea; 3 Sky Labs Inc Seongnam Republic of Korea; 4 Department of Information and Communication Engineering Hanbat National University Daejeon Republic of Korea

**Keywords:** atrial fibrillation, deep learning, diagnosis, photoplethysmography, wearable electronic devices

## Abstract

**Background:**

Continuous photoplethysmography (PPG) monitoring with a wearable device may aid the early detection of atrial fibrillation (AF).

**Objective:**

We aimed to evaluate the diagnostic performance of a ring-type wearable device (CardioTracker, CART), which can detect AF using deep learning analysis of PPG signals.

**Methods:**

Patients with persistent AF who underwent cardioversion were recruited prospectively. We recorded PPG signals at the finger with CART and a conventional pulse oximeter before and after cardioversion over a period of 15 min (each instrument). Cardiologists validated the PPG rhythms with simultaneous single-lead electrocardiography. The PPG data were transmitted to a smartphone wirelessly and analyzed with a deep learning algorithm. We also validated the deep learning algorithm in 20 healthy subjects with sinus rhythm (SR).

**Results:**

In 100 study participants, CART generated a total of 13,038 30-s PPG samples (5850 for SR and 7188 for AF). Using the deep learning algorithm, the diagnostic accuracy, sensitivity, specificity, positive-predictive value, and negative-predictive value were 96.9%, 99.0%, 94.3%, 95.6%, and 98.7%, respectively. Although the diagnostic accuracy decreased with shorter sample lengths, the accuracy was maintained at 94.7% with 10-s measurements. For SR, the specificity decreased with higher variability of peak-to-peak intervals. However, for AF, CART maintained consistent sensitivity regardless of variability. Pulse rates had a lower impact on sensitivity than on specificity. The performance of CART was comparable to that of the conventional device when using a proper threshold. External validation showed that 94.99% (16,529/17,400) of the PPG samples from the control group were correctly identified with SR.

**Conclusions:**

A ring-type wearable device with deep learning analysis of PPG signals could accurately diagnose AF without relying on electrocardiography. With this device, continuous monitoring for AF may be promising in high-risk populations.

**Trial Registration:**

ClinicalTrials.gov NCT04023188; https://clinicaltrials.gov/ct2/show/NCT04023188

## Introduction

Atrial fibrillation (AF) is the most common cardiac arrhythmia, and its prevalence has rapidly increased, especially in the elderly population [1]. In view of this trend, 17.9 million adults are expected to develop AF in Europe by the year 2060 [2]. The socioeconomic burdens of AF are also increasing rapidly in line with its prevalence such that annual medical expenses associated with AF have risen at least five fold in the last decade [3]. Considering the serious complications of AF, early diagnosis and proper management are important.

However, the early detection of AF is challenging owing to its paroxysmal nature [4]. This characteristic makes single electrocardiography (ECG) screening no better than pulse palpation to detect silent AF [5]. Besides, the diagnosis of early AF tends to be delayed because the condition is often asymptomatic [6]. This unmet need for early AF detection may be relieved by continuously monitoring the cardiac rhythm in high-risk populations [7]. However, the cost and invasiveness of an implantable loop recorder limit its use as a continuous monitoring device.

Recently, photoplethysmography (PPG) has been assessed to generate a novel biosignal to monitor AF [8-11]. Compared with ECG, PPG has advantages in terms of accessibility and applicability to wearable or mobile devices [12]. In contrast to an inconvenient traditional strategy to confirm cardiac arrhythmia whereby the patient needs to visit the hospital and undergo ECG, PPG can be easily performed at home using a smartphone or a wearable device. Recently, the Apple Heart Study showed that this strategy has the potential to detect underlying AF in the general population [13]. Moreover, AF detection with PPG can be accurate with deep learning analysis, without relying on ECG [14]. However, the accuracy of PPG depends on the site of measurement [15]. The finger has the highest amplitude and the smallest pulse peak time and reflection index for PPG compared with other body parts; thus, it provides maximum information that can be analyzed [16]. The finger also receives a more abundant supply of arterial blood than the wrist and is easier to affix sensors, hence improving signal quality. As a result, collection of PPG signals from the finger is likely to yield better signal quality than that from the wrist. Therefore, a ring-type wearable device may be a more suitable candidate for the acquisition of PPG signals than a wrist-type wearable device, such as the Apple Watch. Thus, we hypothesized that a ring-type wearable device monitoring PPG data would have high diagnostic performance in the detection of AF. This study aimed to develop a ring-type wearable device (CardioTracker, CART) to detect AF with deep learning analysis of PPG signals and to evaluate its diagnostic performance in patients with AF.

## Methods

### Study Design and Population

This was a prospective, single-center, observational cohort study conducted from 2018 to 2019. The flowchart of this study is illustrated in Multimedia Appendix 1. Adult patients (aged ≥20 years) with persistent AF who were admitted for elective direct-current cardioversion were eligible for the study. The patients were excluded from the study if their cardiac rhythm just before the cardioversion was not AF. The participants who met the eligibility criteria were introduced to the study and enrolled after obtaining informed consent. The recruitment process was consecutive, and measurements were performed in the order of consent to participate in the study.

For direct-current cardioversion, electric shocks of 100-200 J with a biphasic defibrillator were delivered by paddles under light sedation. The cardiac rhythms before and after the cardioversion were validated with 12-lead ECG read by three cardiologists. If there was a discrepancy, a senior electrophysiologist (EKC or EL) assessed the final cardiac rhythm. Both before and after the cardioversion, each participant was at rest in the supine position and PPG and simultaneous single-lead ECG were recorded over 15 min. We did not measure PPG signals after the shock delivery for those in whom cardioversion was unsuccessful. The study protocol was approved by the Institutional Review Board of Seoul National University Hospital and adhered to the Declaration of Helsinki (approval no: 1801-081-916). The study has been registered at ClinicalTrials.gov (NCT04023188).

### Measurements

**Figure 1:**
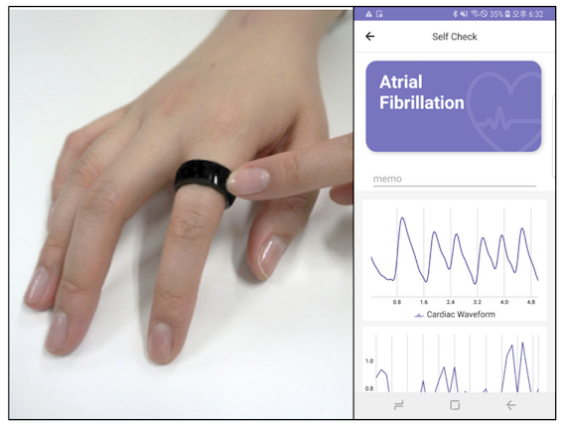
Demonstration of photoplethysmography (PPG) monitoring by CardioTracker (CART). CART measures PPG signals at the proximal phalanx and wirelessly transmits the data to the linked smartphone, which can monitor the PPG signals in real-time, and the deep learning algorithm suggests a possible diagnosis.

This study used PPG measurements by CART (Sky Labs Inc, Seongnam, Republic of Korea) and a conventional medical-grade pulse oximeter (iDAQ-400 with PPG-AMP and P400, PhysioLab Inc, Busan, Republic of Korea) as the two index tests and synchronized single-lead ECG (lead I) as the reference standard. The two devices recorded PPG signals simultaneously (CART at the proximal phalanx and the conventional device at the fingertip). The participant chose a finger that was the most comfortable for PPG measurements and wore CART. Wearing CART was not very different from wearing a conventional ring. However, to ensure proper signal quality, other fingers were selected if there were scars, thick skin, or tremors. Moreover, three different sized CART devices were prepared for proper contact between the skin and PPG sensors. The measurements of both PPG signals and the single-lead ECG were synchronized. The PPG signals from the conventional device and the single-lead ECG were tracked and recorded by monitoring equipment kept at the bedside, whereas the PPG signals from CART were wirelessly transmitted to a research-purpose smartphone in real time (Figure 1). The rhythms of PPG data were confirmed and labeled by reading synchronized single-lead ECG strips. The cardiac rhythms were classified into sinus rhythm (SR) or AF. Here, SR included a case where there existed premature atrial or ventricular beats. During the measurement, the participant was required to lie still in bed to minimize motion artifacts.

We applied a bandpass filter (0.2-18 Hz) to the PPG signals, recorded them at a sampling frequency of 50 Hz, and exported them in XML format for preprocessing. Examples of PPG data from CART and the conventional pulse oximeter are illustrated in Multimedia Appendix 2.

For data augmentation [14,17], each 15-min PPG datum was divided into 30-s samples with 20-s overlaps. For the deep learning process, every sample was labelled as AF or SR according to the rhythm of its synchronized single-lead ECG.

### Ring-Type Wearable Device

CART has been developed to collect and analyze PPG signals from the proximal phalanx. The measurement is based on the reflective method [18], using high-intensity green light-emitting diodes (LEDs) and photodiodes (PDs) embedded inside. It analyzes the PPG signals using a deep learning algorithm with a convolutional neural network (CNN) [17,19], which involves nine neural layers, where the top two are fully connected. The neural network was trained by the Adam optimizer [20]. Dropout and L2 regularization were performed to prevent overfitting [21]. The softmax outputs of the neural network were gently calibrated with temperature scaling to exhibit the diagnostic confidence of SR and AF for every testing PPG sample [22].

To compare the CNN to nondeep learning algorithms, CART also uses a linear-kernel support vector machine (SVM) [23], as it has been shown to have the best diagnostic performance among nondeep learning algorithms [24]. For the features of SVM, we used root mean square of the successive differences of RR intervals with Shannon entropy (RMSSD+ShE) [25], autocorrelation [9], and the ensemble of the previous two (RMSSD+ShE and autocorrelation).

Multimedia Appendix 3 illustrates CART and its wireless charging station. The LEDs and PDs of CART were designed to be located under the finger. CART is made of surgical steel and is waterproof and dustproof (IP58 grade). It collects PPG signals at the proximal phalanx by measuring the reflected lights under the finger. It can monitor PPG signals over 60 hours continuously and can store data for up to 10 hours. It can upload the data via Bluetooth to a mobile or cloud server and can be charged wirelessly with its cradle.

### Diagnostic Performance Analysis

We evaluated the baseline characteristics of the study population, including demographics, comorbidities, use of antiarrhythmic agents and anticoagulants, CHA_2_DS_2_-VASc scores, and history of AF. Multiple five-fold cross-validation processes were used to perform the training and testing processes of the deep learning algorithm. Each validation process randomly assigned 80% of the total participants for training and the other 20% for testing. The validation process was repeated 10 times for each combination of training and testing datasets, resulting in a total of 50 validation processes for evaluating overall diagnostic performance. For the deep learning process, entire 30-s PPG samples were used without pre-extracted features in training or testing. For a given PPG sample, the deep learning algorithm identified SR or AF, whichever had higher diagnostic confidence. There were no indeterminate or missing data in the two index tests and the reference standard.

To evaluate the diagnostic performance of CART according to the length of the PPG samples, we generated 25-, 20-, 15-, 10-, and 5-s PPG samples from the raw data and repeated the analysis. To investigate whether its performance is affected by the variability of peak-to-peak intervals or the pulse rate of PPG signals, we calculated both the coefficient of variation of the peak-to-peak intervals and the pulse rate for every sample. The diagnostic performance according to the variability and pulse rate was evaluated. As the use of a ring-type device for PPG measurement is not fully understood yet, we compared the two index tests (CART and conventional device) by performing the same analysis but with different PPG measurements. We also assessed the accuracy of the diagnostic performance by trying different subsets of PPG samples; a PPG sample was allowed to be tested by the deep learning algorithm only if its diagnostic confidence was higher than a certain threshold level. For external validation of CART with the deep learning algorithm, 20 healthy subjects with SR were additionally recruited. The PPG signals were measured by CART and processed according to the same protocol in each subject. Considering the random characteristics of deep learning, we repeated the testing 10 times.

### Statistical Analysis

The Kolmogorov-Smirnov test was used to check for normal distribution of clinical variables. The data are presented as mean (SD) for age, median with IQR for body mass index and CHA_2_DS_2_-VASc score, or n (%) for other variables. We obtained cross-tabulation from the validation process and calculated the sensitivity, specificity, positive-predictive value (PPV), negative-predictive value (NPV), and diagnostic accuracy (the ratio between the number of correct cases and the total number of tests). A receiver operating characteristic curve was constructed, and the area under the curve (AUC) with 95% CI was calculated using the diagnostic confidence (CNN) or features (SVM). The mean pulse rate was compared between SR and AF samples using the Student *t* test. All statistical analyses were two-sided, and *P*<.05 was considered statistically significant. The data were analyzed using SPSS version 22.0 (IBM Corp, Armonk, New York, USA).

## Results

### Baseline Characteristics

The baseline characteristics of the study population are illustrated in Table 1. A total of 100 participants (81 male participants, 81%; mean age 63.8 years, SD 8.5; median CHA_2_DS_2_-VASc score 2) were enrolled in this study. We collected a total of 13,038 30-s PPG samples (5850 for SR and 7188 for AF) from this population, using CART. Among the 100 participants, 81 had persistent AF and the other 19 had long-standing persistent AF. In 15 participants, cardioversion was unsuccessful. The mean pulse rate was higher in AF samples than in SR samples (63.5, SD 9.9 vs 59.6, SD 9.9; *P*<.001). There were no adverse events or safety issues during the study.

**Table 1:** Baseline characteristics of the study population (N=100).

Characteristic		Value^a^
**Demographics**		
	Age (years)	63.8 (8.5)
	Male	81 (81.0)
	Median body mass index (kg/m^2^)	25.3 (23.5-27.1)
	Median CHA_2_DS_2_-VASc score	2 (1-3)
	Atrial fibrillation ablation history	7 (7.0)
**Types of AF**		
	Persistent^b^	81 (81.0)
	Long-standing persistent^c^	19 (19.0)
**Comorbidity**		
	Congestive heart failure	15 (15.0)
	Hypertension	57 (57.0)
	Diabetes mellitus	27 (27.0)
	Stroke or transient ischemic attack	4 (4.0)
	Myocardial infarction or ischemic heart disease	6 (6.0)
	Valvular heart disease	3 (3.0)
	Dyslipidemia	35 (35.0)
	Chronic renal failure	3 (3.0)
	Chronic obstructive pulmonary disease	1 (1.0)
	Hyperthyroidism	3 (3.0)
**Antiarrhythmic agents**		
	Propafenone	17 (17.0)
	Flecainide	10 (10.0)
	Pilsicainide	3 (3.0)
	Sotalol	0 (0)
	Amiodarone	64 (64.0)
	Beta-blocker	24 (24.0)
	Nondihydropyridine calcium channel blocker	27 (27.0)
	Digoxin	2 (2.0)
**Anticoagulants**		
	Warfarin	9 (9.0)
	Nonvitamin K oral anticoagulant	91 (91.0)
**Other medications**		
	Angiotensin-converting enzyme inhibitor	100 (100)
	Angiotensin II receptor blocker	29 (29.0)
	Diuretics	15 (15.0)
	Statin	32 (32.0)

^a^Values are mean (SD) for age, median (IQR) for body mass index and CHA_2_DS_2_-VASc score, or n (%) for other variables.

^b^Atrial fibrillation history for more than 1 month but less than 1 year.

^c^Atrial fibrillation history for more than 1 year.

### Diagnostic Performance According to the Algorithms

**Figure 2:**
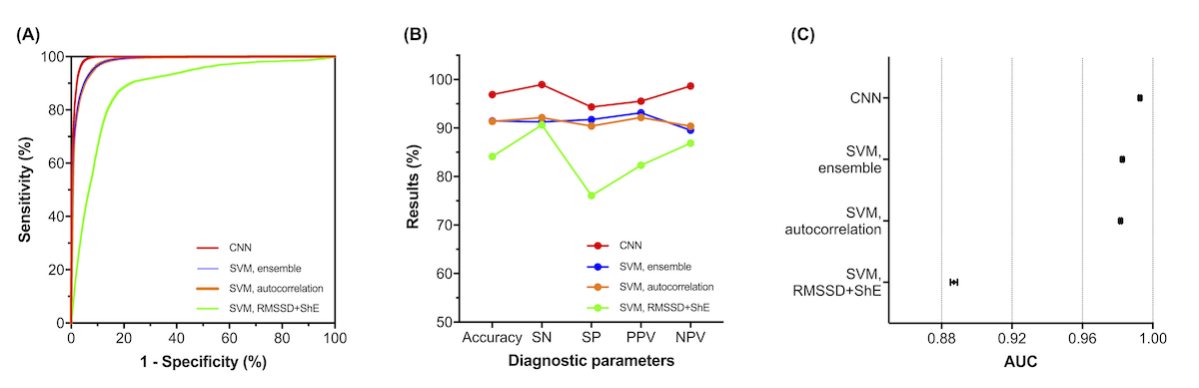
Diagnostic performance of CardioTracker (CART) according to the algorithms. CART with the deep learning algorithm achieved the highest results for all diagnostic parameters. (A) ROC curves, (B) Diagnostic parameters, and (C) AUCs according to the algorithms. AUC: area under the curve, CNN: convolutional neural network, NPV: negative-predictive value, PPV: positive-predictive value, ROC: receiver operating characteristic, SN: sensitivity, SP: specificity, SVM, autocorrelation: support vector machine with autocorrelation as a feature, SVM, RMSSD+ShE: support vector machine with root mean square of the successive differences of RR intervals and Shannon entropy as features, SVM, ensemble: support vector machine with all three features.

The performance of CART according to the algorithms is presented in Table 2 and Figure 2. Combined with the CNN algorithm, it showed the highest performance for all the diagnostic parameters, with diagnostic accuracy of 96.89%, sensitivity of 98.96%, specificity of 94.34%, PPV of 95.55%, NPV of 98.67%, and AUC (95% CI) of 0.993 (0.992-0.993). Among the nondeep learning algorithms, SVM with the ensemble method had the highest results for all the parameters, except sensitivity and NPV, with diagnostic accuracy of 91.49%, sensitivity of 91.29%, specificity of 91.74%, PPV of 93.14%, NPV of 89.55%, and AUC (95% CI) of 0.983 (0.982-0.983). Adding RMSSD and ShE to autocorrelation as features did not significantly improve the performance of SVM (Figure 2).

**Table 2:** Diagnostic performance of the ring according to algorithms.

Algorithm	Accuracy, mean percentage	Sensitivity, mean percentage	Specificity, mean percentage	Positive-predictive value, mean percentage	Negative-predictive value, mean percentage	AUC^a^ (95% CI)
Convolutional neural network	96.89	98.96	94.34	95.55	98.67	0.993 (0.992-0.993)
SVM^b^, ensemble^c^	91.49	91.29	91.74	93.14	89.55	0.983 (0.982-0.983)
SVM, autocorrelation^d^	91.37	92.15	90.4	92.18	90.36	0.982 (0.981-0.982)
SVM, RMSSD^e^+ShE^f g^	84.11	90.65	76.07	82.31	86.88	0.887 (0.885-0.889)

^a^AUC: area under the receiver operating characteristic curve.

^b^SVM: support vector machine.

^c^SVM with autocorrelation, RMSSD, and ShE as features.

^d^SVM with autocorrelation as a feature.

^e^RMSSD: root mean square of the successive differences of RR intervals.

^f^ShE: Shannon entropy.

^g^SVM with RMSSD and ShE as features.

### Impact of Sample Length

**Figure 3:**
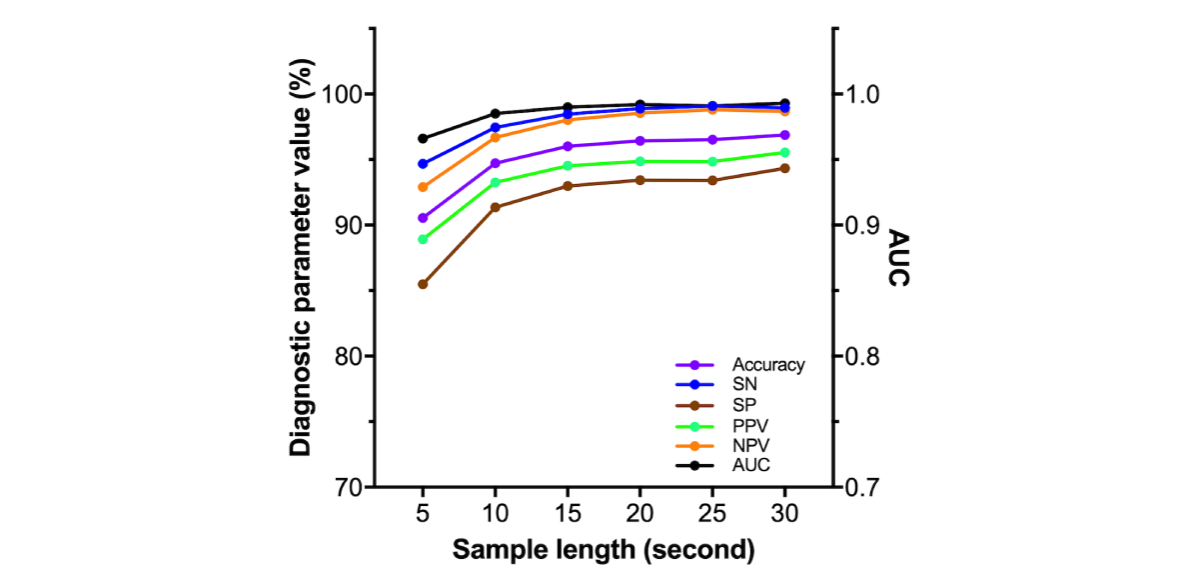
The diagnostic performance of CardioTracker according to sample length. In general, longer lengths of photoplethysmography samples had higher diagnostic performances. AUC: area under the curve, NPV: negative-predictive value, PPV: positive-predictive value, SN: sensitivity, SP: specificity.

The association between sample length and the diagnostic performance of CART is presented in Figure 3 and Table 3. The figure only presents the results of CART with CNN. As expected, all diagnostic parameters decreased as sample length shortened. Using 10-s PPG segments, CART achieved diagnostic accuracy of 94.72%, sensitivity of 97.46%, specificity of 91.35%, PPV of 93.26%, NPV of 96.69%, and AUC (95% CI) of 0.985 (0.985-0.986).

**Table 3:** Diagnostic performance according to sample length.

Duration (s)	Accuracy, mean percentage	Sensitivity, mean percentage	Specificity, mean percentage	Positive-predictive value, mean percentage	Negative-predictive value, mean percentage	AUC^a^ (95% CI)
30	96.89	98.96	94.34	95.55	98.67	0.993 (0.992-0.993)
25	96.53	99.08	93.40	94.85	98.80	0.991 (0.990-0.991)
20	96.44	98.89	93.43	94.87	98.56	0.992 (0.992-0.992)
15	96.01	98.47	92.99	94.52	98.02	0.990 (0.990-0.990)
10	94.72	97.46	91.35	93.26	96.69	0.985 (0.985-0.986)
5	90.55	94.68	85.49	88.91	92.90	0.966 (0.965-0.966)

^a^AUC: area under the receiver operating characteristic curve.

### Impact of Premature Beats

**Figure 4:**
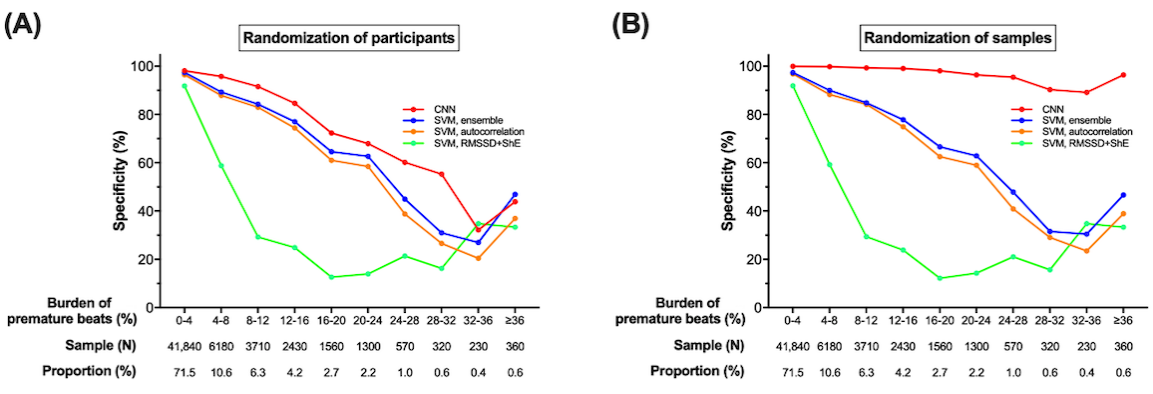
The specificity of CardioTracker according to the burden of premature beats. (A) The five-fold cross-validation process with randomization of participants. There was a decreasing trend of specificity according to increasing burden of premature beats. However, the convolutional neural network (CNN) maintained the highest results for most cases. (B) The five-fold cross-validation process with randomization of samples. The CNN improved specificity in especially high burden of premature beats. SVM, autocorrelation: support vector machine with autocorrelation as a feature, SVM, RMSSD+ShE: support vector machine with root mean square of the successive differences of RR intervals and Shannon entropy as features, SVM, ensemble: support vector machine with all three features.

Each SR-labelled PPG sample may have a record of atrial or ventricular premature beats, which can be confirmed by evaluating synchronized ECG. We evaluated the specificity of our device by assessing the burden of premature beats (Figure 4). The total number of samples was 10 times the number of SR samples (58,500), as the validation processes were repeated 10 times. When participants were randomized for the five-fold cross-validation processes such that the algorithm always encountered new participants in the testing, higher burdens of premature beats deteriorated the specificity of CART, regardless of the algorithm. Among the algorithms, CNN maintained the highest results for most cases of premature beat burdens. When samples were randomized such that the algorithm might encounter the same participants in testing, there was an improvement in CNN performance, especially for higher burdens of premature beats, and CNN maintained overall consistent results, regardless of the burden. As this validation process simulates a situation with a sufficiently large number of participants in the training dataset, this finding implies that the performance would improve with an increasing population, regardless of the burden of premature beats.

### Impact of the Variability of Peak-to-Peak Intervals and the Pulse Rate

**Figure 5:**
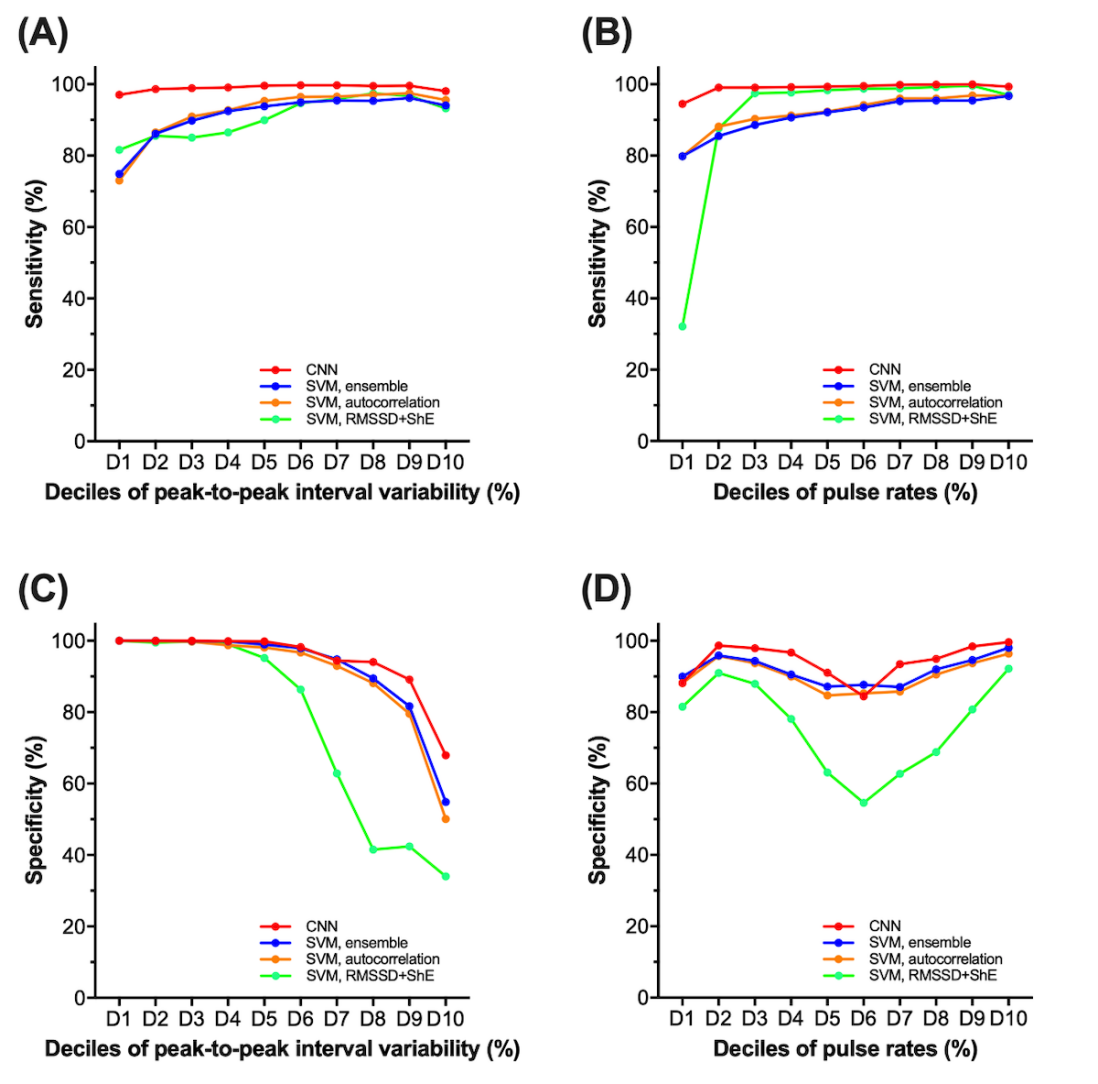
The sensitivity and specificity of CardioTracker according to the characteristics of samples. (A) and (B) With the deep learning algorithm, there were no definite associations between the sensitivity and peak-to-peak interval variability or the pulse rate. (C) The specificity generally decreased with higher peak-to-peak interval variability. (D) There was generally a U-shape association between specificity and the pulse rate. CNN: convolutional neural network, SVM, autocorrelation: support vector machine with autocorrelation as a feature, SVM, RMSSD+ShE: support vector machine with root mean square of the successive differences of RR intervals and Shannon entropy as features, SVM, ensemble: support vector machine with all three features.

The performance of CART can be affected by the characteristics of the PPG samples. We evaluated whether the variability of peak-to-peak intervals and the pulse rate affected the performance (Figure 5). For sensitivity, higher peak-to-peak interval variability and faster pulse rates were associated with higher sensitivity for SVM. However, for CNN, neither peak-to-peak interval variability nor pulse rate had such a relevant association with sensitivity. This finding suggests that CART with a deep learning algorithm is less affected by peak-to-peak interval variability or the pulse rate of AF.

For specificity, the performance of CART decreased with higher peak-to-peak interval variability regardless of the algorithm. However, for CNN, only the extremes of the variability (the ninth and the tenth deciles) had decreased specificity less than 90%. This finding was expected, as SR with higher peak-to-peak interval variability mimics AF to a great extent. There was a nonlinear association between specificity and pulse rate, and in general, the results were the highest with CNN. The complicated association between specificity and pulse rate can be mostly explained by evaluating the association between the burden of premature beats and the pulse rate (Multimedia Appendix 4). For example, lower specificity for the sixth decile of the pulse rate can be due to the higher burden of premature beats.

### Visualization of Deep Learning Analyses

**Figure 6:**
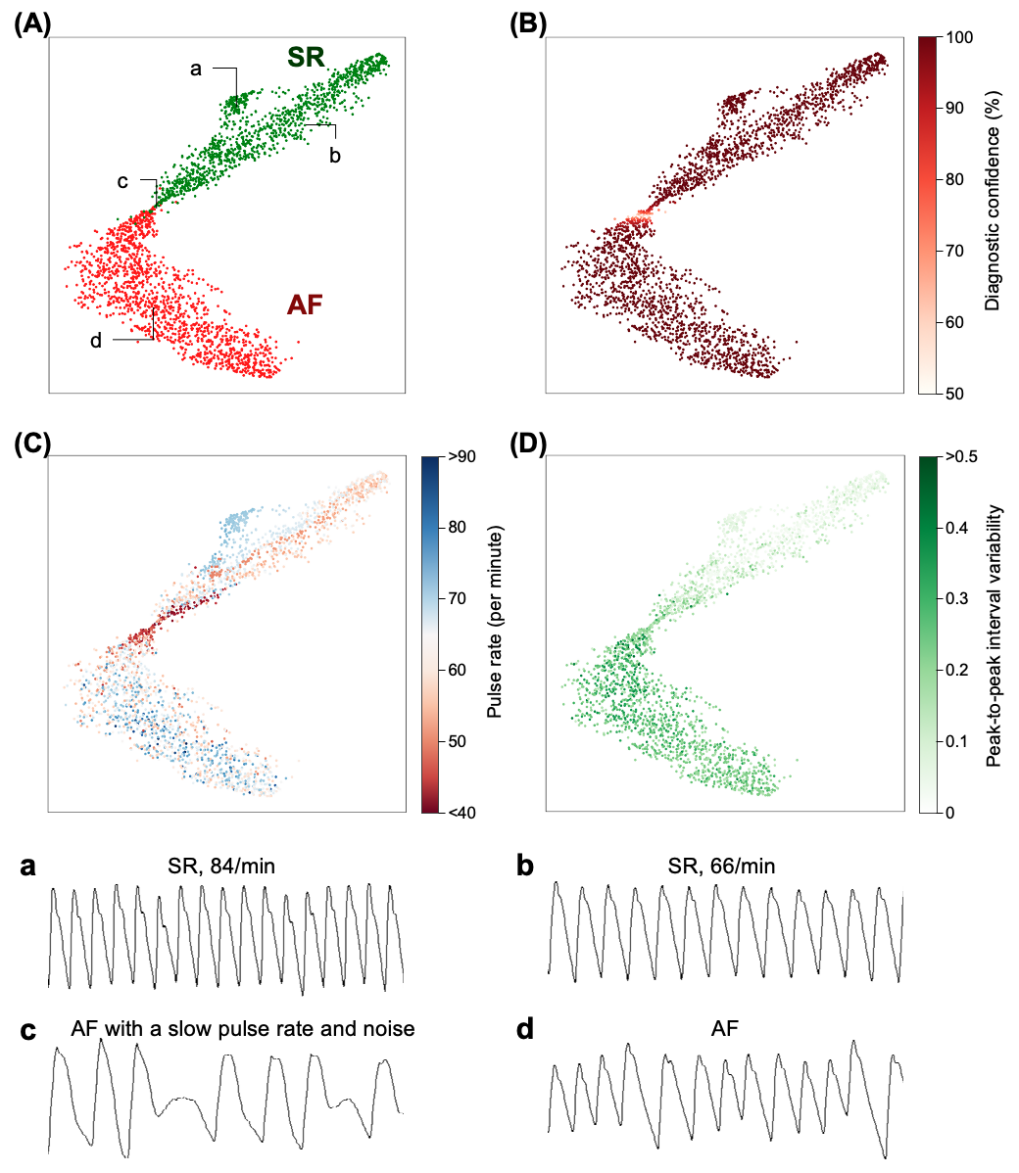
Visualization of deep learning analyses. The deep learning analyses of CardioTracker are plotted with the t-SNE method. The upper panel: (A) The two clusters of AF and SR were well differentiated from each other, leaving a small overlapped potion. (B), (C), and (D) The overlapped region showed low diagnostic confidence, low pulse rates, and modest peak-to-peak interval variability. The lower panel: typical examples of photoplethysmography samples. AF: atrial fibrillation, SR: sinus rhythm, t-SNE: t-distributed stochastic neighbor embedding.

The deep learning analyses for CART are illustrated in Figure 6 by mapping extracted features from the deep learning algorithm into two-dimensional space. According to the t-distributed stochastic neighbor embedding plot, the cluster of AF was well differentiated from the counterpart of SR. In the region where the two clusters overlapped, lower diagnostic confidences were observed, which suggests that the deep learning algorithm mostly failed when the PPG samples belonged to this region. When we applied heatmaps with the pulse rate and peak-to-peak interval variability, this region had characteristics with lower pulse rates and modest variabilities. When we inspected the actual PPG data, this region also exhibited noisy signals. Therefore, PPG samples with lower pulse rates, modest variabilities, and noise would likely have low diagnostic performance. The cluster of AF was homogeneous in terms of the pulse rate, whereas the cluster of SR had distinctive subportions according to the actual pulse rate.

### CardioTracker and the Conventional Pulse Oximeter

We evaluated the two index tests in parallel and observed the changes in diagnostic performances according to the threshold level of diagnostic confidence. Compared with the conventional pulse oximeter, CART showed comparable diagnostic performance (Table 4). In both devices, all the diagnostic parameters improved with increasing threshold levels (Multimedia Appendix 5). This finding is expected, as the diagnosis would become more accurate for samples with higher diagnostic confidence. However, this improvement was counter-balanced by increasing the proportion of filtered samples (not tested by the deep learning algorithm). From these findings, CART appears to be comparable to the conventional pulse oximeter when used as a PPG measurement device. Moreover, the performance of CART can be tuned by applying different threshold levels.

**Table 4:** Comparison of diagnostic performance between CardioTracker and the conventional pulse oximeter at the fingertip (control).

Device	Accuracy, mean percentage	Sensitivity, mean percentage	Specificity, mean percentage	Positive-predictive value, mean percentage	Negative-predictive value, mean percentage	AUC^a^ (95% CI)^b^
CART^c^	96.89	98.96	94.34	95.55	98.67	0.993 (0.992-0.993)
Control	97.50	99.66	94.89	95.95	99.56	0.995 (0.995-0.995)

^a^AUC: area under the receiver operating characteristic curve.

^b^The standard error by the binomial exact test was less than 0.01.

^c^CART: CardioTracker.

### External Validation of CardioTracker in Healthy Subjects

A total of 1740 PPG samples were obtained from 20 healthy subjects with SR. Among these samples with repeated testing 10 times, 94.99% (16,529/17,400) of the testing cases were correctly identified with SR.

## Discussion

### Principal Findings

This prospective observational cohort study evaluated the diagnostic performance of a ring-type wearable device (CART) to detect AF. To the best of our knowledge, this is the first clinical study to analyze the performance of a ring-type wearable device designed for detecting AF with PPG. The study had several findings. First, we found that the deep learning algorithm can maximize the performance of CART solely based on PPG. Second, a PPG measurement period of about 10 s may be sufficient to detect AF. Third, the data from a sufficiently large number of participants may further improve the performance of CART by enhancing the deep learning process, especially for difficult cases in which the high burden of premature beats mimics AF. Fourth, among the diagnostic parameters, sensitivity may be maintained at a consistently high level regardless of the variability of peak-to-peak intervals or the pulse rate of PPG signals. Fifth, although CART measures PPG at the middle of the finger, which is not the location where a conventional pulse oximeter measures the impulse, its performance is comparable to that of a conventional device. Lastly, we performed external validation of CART with the deep learning algorithm in healthy subjects and observed that the CNN algorithm can diagnose SR accurately.

### Screening for Atrial Fibrillation in a High-Risk Population

AF is known to cause about 10% of the total cases of stroke, and it has been shown to increase the risk of stroke even when discovered incidentally through screening [26]. AF screening is beneficial in an appropriate setting for patients in all localities. Therefore, early diagnosis of AF with appropriate anticoagulant therapy is expected to reduce the risk of ischemic stroke. However, early diagnosis is challenging because paroxysmal or asymptomatic episodes are common. Therefore, further research is needed to find more convenient and effective screening methods. Based on this aspect, PPG has recently attracted attention as a method of AF screening because it can be continuously monitored with appropriate equipment and its measurement is convenient.

### Utility of Photoplethysmography to Detect Atrial Fibrillation

This study used PPG signals measured from CART to detect AF. As there is a good correlation between each pulse of PPG and the corresponding QRS complex on ECG, it is feasible to diagnose AF with PPG. Considering that PPG has limited capability to detect atrial electrical activity, many PPG algorithms have been studied to detect AF using the randomness of peak-to-peak intervals [27,28]. Two issues should be addressed. First, there is uncertainty as to the choice of algorithm used to detect AF. For detecting AF with PPG, deep learning algorithms have been known to achieve the highest diagnostic results so far [14]. The possible reason is that nondeep learning algorithms use only specific data features, which are invented by humans, whereas deep learning algorithms analyze the entire dataset without human guidance. Second, the optimal anatomical location for PPG measurement is debatable. Multiple studies have evaluated the diagnostic value of PPG measured at various sites, including the wrist and face [8,10,12]. However, a recent study showed that the finger has the highest quality of PPG signals [16]. Therefore, to diagnose AF more effectively, it is probably best to analyze PPG signals from the finger. In summary, the most effective AF diagnosis is possible when analyzing PPG signals from the finger and using deep learning algorithms.

### Wearable Devices to Detect Atrial Fibrillation With Photoplethysmography

The method for screening AF should be not only supported by sufficient diagnostic precision but also convenient for patients. The strategy of carrying a portable device involving point-of-care testing is not only inconvenient but also ineffective in that it can miss the diagnosis when AF is asymptomatic. In this context, a wearable device that continuously monitors PPG signals in the background without user intervention would be easy to use and efficient for diagnosis. If CART is worn all day, PPG signals can be continuously monitored; therefore, more AF episodes could be detected. However, continuous monitoring might increase the chance of collecting other signals, such as noise caused by movements in daily life, leading to a decrease in the accuracy of diagnosis. To resolve this problem, we need engineering technology for estimating and correcting motion artifacts through the use of accelerometer sensors in addition to PPG signal analysis. Second, various atrial tachyarrhythmia episodes other than AF could be detected more frequently, which might lower the diagnostic performance for AF detection by CART. Sufficient data should be collected for various atrial tachyarrhythmias in addition to AF to improve machine learning analyses.

Besides, when PPG signals are continuously monitored, various atrial tachyarrhythmia episodes can occur in addition to AF, which may lower the diagnostic performance of CART. If atrial tachyarrhythmia episodes occur frequently, the irregularity of the PPG signal is expected to be similar to that for AF, and in this case, the diagnostic performance of CART may deteriorate. To solve this issue, sufficient data should be collected for various arrhythmias in addition to AF to improve machine learning analyses.

Although wearable devices may not have become popular due to their availability and cost-effectiveness, the usefulness of such devices in the detection of AF has been studied [29]. The wrist-type device is one of the most widely studied wearable devices [13,30,31]. However, the WATCH AF Trial also reported that a high proportion (22%) of PPG signals from smartwatches had an insufficient signal quality for evaluation [31]. Therefore, measuring PPG signals on the wrist may lead to poor signal quality. In order to resolve this issue, other types of wearable devices are necessary, and the ring-type device, which measures PPG signals from the finger, might be ideal. Therefore, a ring-type wearable device could be useful as a new diagnostic tool for high-risk populations in the future.

### Limitations

There are some limitations in this study. First, noise in PPG signals, such as motion artifacts, might affect the analysis. However, motion artifacts were minimized as every participant was required to lie still on the bed during the PPG measurement. The diagnostic performance of CART in an ambulatory setting will be tested, but a sophisticated deep learning algorithm should be developed beforehand to deal with motion artifacts. Second, the performance of CART was not assessed for other arrhythmias. Future studies should analyze the diagnosis of other arrhythmias using PPG signals. Third, the duration of monitoring was relatively short. Longer monitoring times would allow further deep learning training and subsequently yield better results than our results. Fourth, we did not compare performance between CART and other commercially available wrist-type wearable devices. Further studies will provide insights into this issue. Fifth, economic assessment of CART cannot be performed yet. However, in the case of AliveCor, it was shown that a wearable device could be cost-effective for AF screening [29]. Likewise, CART is also expected to reduce the economic burden of diagnosing AF if its market price is reasonable. This economic evaluation requires further research. Sixth, since a 20-s overlap existed between consecutive samples obtained from a subject during data augmentation, it is possible that even if different samples existed in the training and testing datasets, some sections were the same, and thus, the diagnostic performance was improved. Seventh, AF diagnosis by PPG only has limitations. Adding the on-demand recording function of single-lead ECG, similar to an Apple Watch, to CART may compensate for the limitations that arise in diagnoses based on PPG signals. In this case, if AF is suspected during PPG monitoring, a notification can be sent to the user to check the electrocardiogram, so that the user can more clearly check for AF. The validation of such a function would be performed in future research. Lastly, even though there were no adverse events of CART, potential safety issues in long-term use should be addressed in a subsequent study.

### Conclusions

In this study, we validated the performance of a ring-type wearable device (CART) to diagnose AF using PPG signals. The deep learning algorithm aimed to analyze PPG rhythms and suggested a dichotomous diagnosis of either AF or SR. CART with deep learning analysis of PPG signals had good diagnostic performance without relying on ECG. Moreover, as a PPG measurement device, CART generated results comparable to those of a conventional medical-grade pulse oximeter. This new device may be promising for the detection of AF in high-risk or asymptomatic populations.

## Appendix

Multimedia Appendix 1 Study flowchart. AF: atrial fibrillation, CNN: convolutional neural network, CV: cardioversion, ECG: electrocardiography, PPG: photoplethysmography, SVM: support vector machine.

Multimedia Appendix 2 The first row of the figure shows the 15-min measurements from a subject with the single-lead ECG, the PPG from the conventional pulse oximeter, and the PPG from CardioTracker. The second row of the figure shows one of the 30-s fractions of the raw data. The third row of the figure shows the preprocessed 30-s sample of the PPG signals obtained from the two devices. ECG: electrocardiography, PPG: photoplethysmography.

Multimedia Appendix 3 The left figure illustrates CardioTracker (CART) and its wireless charging station. The right figure shows the position of the light-emitting diode (LED) and photodiode (PD) of CART.

Multimedia Appendix 4 The burden of premature beats according to the deciles of the pulse rate. Each error bar represents the 95% CI of the corresponding burden of premature beats.

Multimedia Appendix 5 The performances of CardioTracker and a conventional pulse oximeter according to threshold levels of diagnostic probability. Both devices showed improved performance with increasing threshold levels. NPV: negative-predictive value, PPV: positive-predictive value.

## Abbreviations

AF: atrial fibrillation
AUC: area under the curve
CART: CardioTracker
CNN: convolutional neural network
ECG: electrocardiography
NPV: negative-predictive value
PPG: photoplethysmography
PPV: positive-predictive value
RMSSD: root mean square of the successive differences of RR intervals
ShE: Shannon entropy
SR: sinus rhythm
SVM: support vector machine

## References

[1] Lee S, Choi E, Han K, Cha M, Oh S (2017). Trends in the incidence and prevalence of atrial fibrillation and estimated thromboembolic risk using the CHADS-VASc score in the entire Korean population. Int J Cardiol.

[2] Krijthe BP, Kunst A, Benjamin EJ, Lip GY, Franco OH, Hofman A, Witteman JC, Stricker BH, Heeringa J (2013). Projections on the number of individuals with atrial fibrillation in the European Union, from 2000 to 2060. Eur Heart J.

[3] Lee H, Kim T, Baek Y, Uhm J, Pak H, Lee M, Joung B (2017). The Trends of Atrial Fibrillation-Related Hospital Visit and Cost, Treatment Pattern and Mortality in Korea: 10-Year Nationwide Sample Cohort Data. Korean Circ J.

[4] Wyse DG, Van Gelder IC, Ellinor PT, Go AS, Kalman JM, Narayan SM, Nattel S, Schotten U, Rienstra M (2014). Lone atrial fibrillation: does it exist?. J Am Coll Cardiol.

[5] Heidt ST, Kratz A, Najarian K, Hassett AL, Oral H, Gonzalez R, Nallamothu BK, Clauw D, Ghanbari H (2016). Symptoms In Atrial Fibrillation: A Contemporary Review And Future Directions. J Atr Fibrillation.

[6] Jonas DE, Kahwati LC, Yun JD, Middleton JC, Coker-Schwimmer M, Asher GN (2018). Screening for Atrial Fibrillation With Electrocardiography: Evidence Report and Systematic Review for the US Preventive Services Task Force. JAMA.

[7] Camm AJ (2014). The Role of Continuous Monitoring in Atrial Fibrillation Management. Arrhythm Electrophysiol Rev.

[8] Yan BP, Lai WH, Chan CK, Chan SC, Chan L, Lam K, Lau H, Ng C, Tai L, Yip K, To OT, Freedman B, Poh YC, Poh M (2018). Contact-Free Screening of Atrial Fibrillation by a Smartphone Using Facial Pulsatile Photoplethysmographic Signals. J Am Heart Assoc.

[9] Chan P, Wong C, Poh YC, Pun L, Leung WW, Wong Y, Wong MM, Poh M, Chu DW, Siu C (2016). Diagnostic Performance of a Smartphone-Based Photoplethysmographic Application for Atrial Fibrillation Screening in a Primary Care Setting. J Am Heart Assoc.

[10] Conroy T, Guzman JH, Hall B, Tsouri G, Couderc J (2017). Detection of atrial fibrillation using an earlobe photoplethysmographic sensor. Physiol Meas.

[11] Mc MD, Chong JW, Soni A, Saczynski JS, Esa N, Napolitano C, Darling CE, Boyer E, Rosen RK, Floyd KC, Chon KH (2016). PULSE-SMART: Pulse-Based Arrhythmia Discrimination Using a Novel Smartphone Application. J Cardiovasc Electrophysiol.

[12] Bonomi AG, Schipper F, Eerikäinen LM, Margarito J, van Dinther R, Muesch G, de Morree HM, Aarts RM, Babaeizadeh S, McManus DD, Dekker LR (2018). Atrial Fibrillation Detection Using a Novel Cardiac Ambulatory Monitor Based on Photo-Plethysmography at the Wrist. J Am Heart Assoc.

[13] Perez MV, Mahaffey KW, Hedlin H, Rumsfeld JS, Garcia A, Ferris T, Balasubramanian V, Russo AM, Rajmane A, Cheung L, Hung G, Lee J, Kowey P, Talati N, Nag D, Gummidipundi SE, Beatty A, Hills MT, Desai S, Granger CB, Desai M, Turakhia MP, Apple Heart Study Investigators (2019). Large-Scale Assessment of a Smartwatch to Identify Atrial Fibrillation. N Engl J Med.

[14] Kwon S, Hong J, Choi E, Lee E, Hostallero DE, Kang WJ, Lee B, Jeong E, Koo B, Oh S, Yi Y (2019). Deep Learning Approaches to Detect Atrial Fibrillation Using Photoplethysmographic Signals: Algorithms Development Study. JMIR Mhealth Uhealth.

[15] Nilsson L, Goscinski T, Kalman S, Lindberg L, Johansson A (2007). Combined photoplethysmographic monitoring of respiration rate and pulse: a comparison between different measurement sites in spontaneously breathing subjects. Acta Anaesthesiol Scand.

[16] Hartmann V, Liu H, Chen F, Qiu Q, Hughes S, Zheng D (2019). Quantitative Comparison of Photoplethysmographic Waveform Characteristics: Effect of Measurement Site. Front Physiol.

[17] Krizhevsky A, Sutskever I, Hinton GE (2012). ImageNet classification with deep convolutional neural networks.

[18] Tamura T, Maeda Y, Sekine M, Yoshida M (2014). Wearable Photoplethysmographic Sensors—Past and Present. Electronics.

[19] Zhang X, Zhou X, Lin M, Sun J (2018). ShuffleNet: An Extremely Efficient Convolutional Neural Network for Mobile Devices. Proceedings of IEEE Computer Society Conference on Computer Vision and Pattern Recognition.

[20] Kingma DP, Ba JL (2014). Adam: A method for stochastic optimization. arXiv:14126980.

[21] Srivastava N, Hinton G, Krizhevsky A, Sutskever I, Salakhutdinov R (2014). Dropout: a simple way to prevent neural networks from overfitting. The Journal of Machine Learning Research.

[22] Guo C, Pleiss G, Sun Y, Weinberger KQ (2017). On Calibration of Modern Neural Networks. arXiv:170604599.

[23] Suykens J, Vandewalle J (1999). Least Squares Support Vector Machine Classifiers. Neural Processing Letters.

[24] Poh M, Poh YC, Chan P, Wong C, Pun L, Leung WW, Wong Y, Wong MM, Chu DW, Siu C (2018). Diagnostic assessment of a deep learning system for detecting atrial fibrillation in pulse waveforms. Heart.

[25] Lee K, Choi HO, Min SD, Lee J, Gupta BB, Nam Y (2017). A Comparative Evaluation of Atrial Fibrillation Detection Methods in Koreans Based on Optical Recordings Using a Smartphone. IEEE Access.

[26] Freedman B, Camm J, Calkins H, Healey JS, Rosenqvist M, Wang J, Albert CM, Anderson CS, Antoniou S, Benjamin EJ, Boriani G, Brachmann J, Brandes A, Chao T, Conen D, Engdahl J, Fauchier L, Fitzmaurice DA, Friberg L, Gersh BJ, Gladstone DJ, Glotzer TV, Gwynne K, Hankey GJ, Harbison J, Hillis GS, Hills MT, Kamel H, Kirchhof P, Kowey PR, Krieger D, Lee VW, Levin L, Lip GY, Lobban T, Lowres N, Mairesse GH, Martinez C, Neubeck L, Orchard J, Piccini JP, Poppe K, Potpara TS, Puererfellner H, Rienstra M, Sandhu RK, Schnabel RB, Siu C, Steinhubl S, Svendsen JH, Svennberg E, Themistoclakis S, Tieleman RG, Turakhia MP, Tveit A, Uittenbogaart SB, Van Gelder IC, Verma A, Wachter R, Yan BP, AF-Screen Collaborators (2017). Screening for Atrial Fibrillation: A Report of the AF-SCREEN International Collaboration. Circulation.

[27] Shan SM, Tang SC, Huang PW, Lin YM, Huang WH, Lai DM, Wu AY (2016). Reliable PPG-based algorithm in atrial fibrillation detection.

[28] Chong JW, Esa N, McManus DD, Chon KH (2015). Arrhythmia discrimination using a smart phone. IEEE J Biomed Health Inform.

[29] Giebel GD, Gissel C (2019). Accuracy of mHealth Devices for Atrial Fibrillation Screening: Systematic Review. JMIR Mhealth Uhealth.

[30] Tison GH, Sanchez JM, Ballinger B, Singh A, Olgin JE, Pletcher MJ, Vittinghoff E, Lee ES, Fan SM, Gladstone RA, Mikell C, Sohoni N, Hsieh J, Marcus GM (2018). Passive Detection of Atrial Fibrillation Using a Commercially Available Smartwatch. JAMA Cardiol.

[31] Dörr M, Nohturfft V, Brasier N, Bosshard E, Djurdjevic A, Gross S, Raichle CJ, Rhinisperger M, Stöckli R, Eckstein J (2019). The WATCH AF Trial: SmartWATCHes for Detection of Atrial Fibrillation. JACC Clin Electrophysiol.

