# Supplementary figures and images for: Detection of Atrial Fibrillation Using a Ring-Type Wearable Device (CardioTracker) and Deep Learning Analysis of Photoplethysmography Signals: Prospective Observational Proof-of-Concept Study
Source: J Med Internet Res. 2020 May 21;22(5):e16443. doi: 10.2196/16443 (PMC7273241; doi:10.2196/16443)

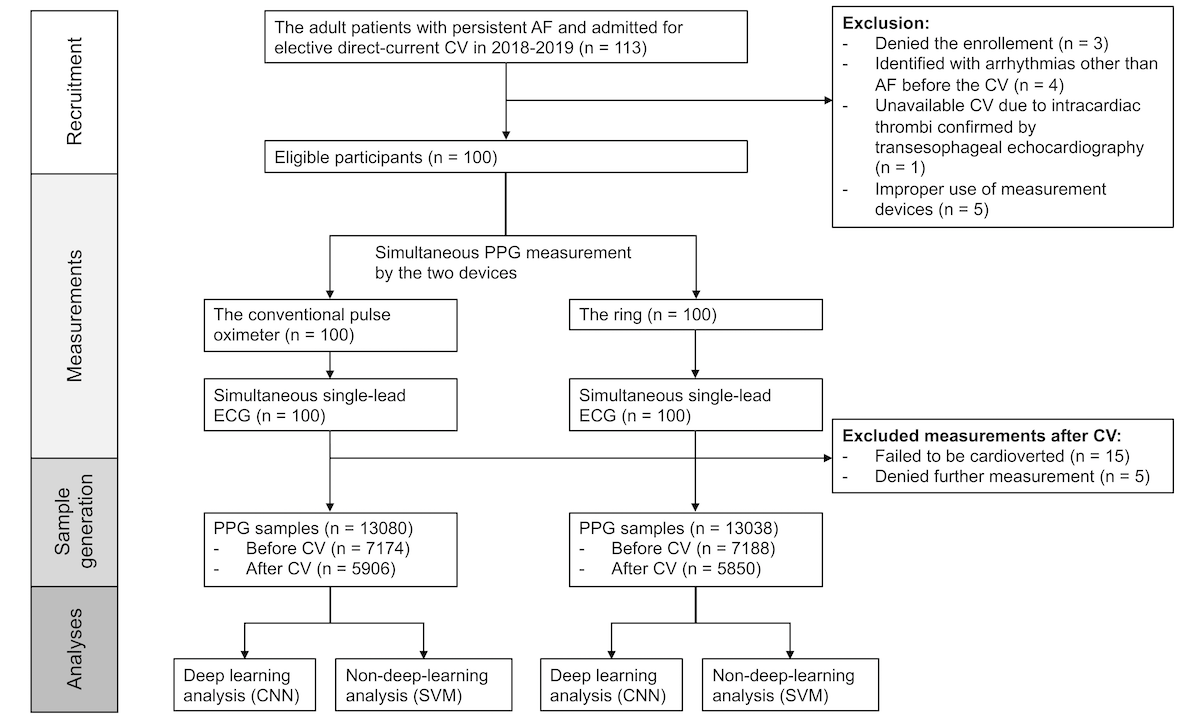

Supplement: Multimedia Appendix 1 [file jmir_v22i5e16443_app1.png]

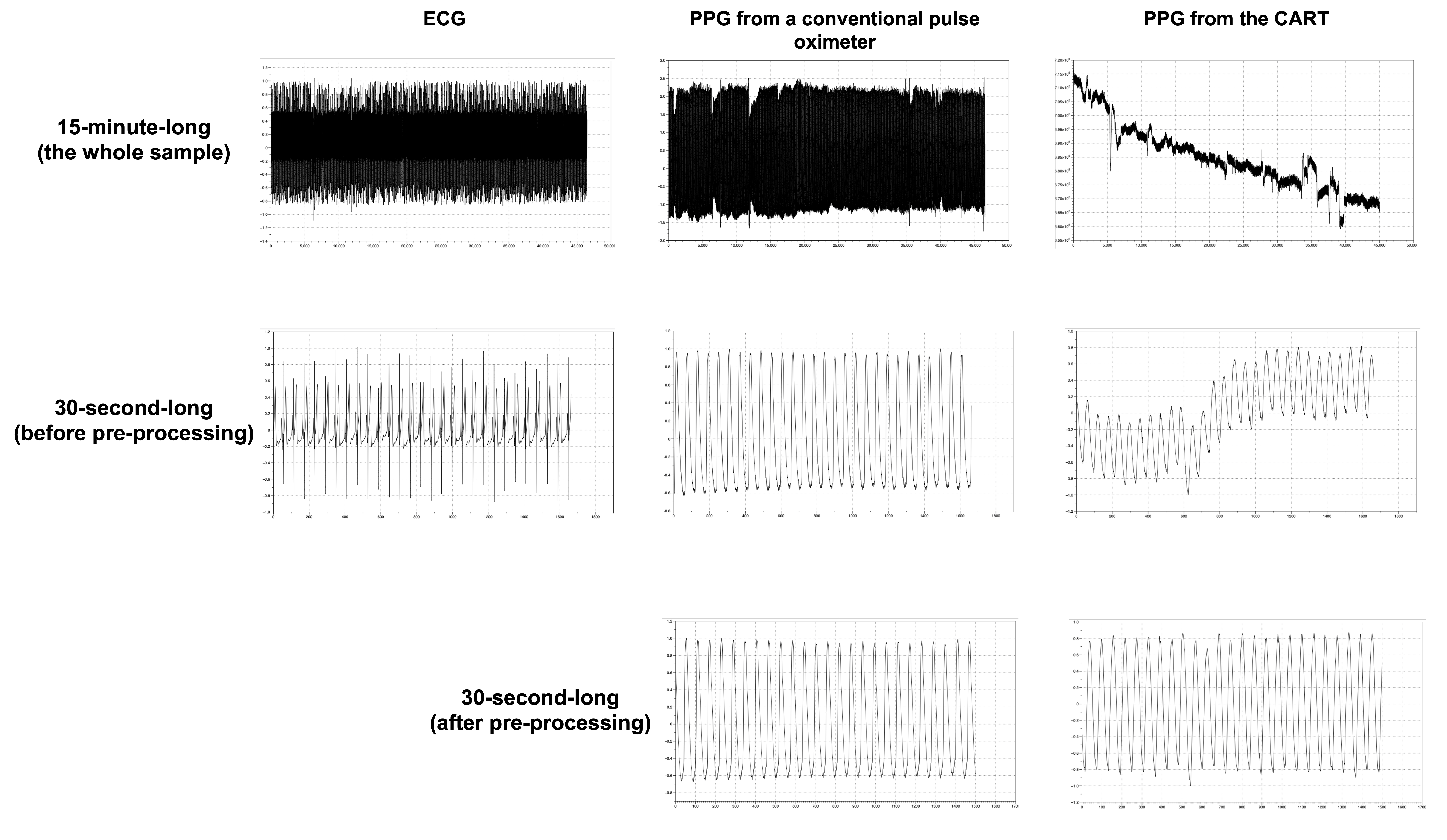

Supplement: Multimedia Appendix 2 [file jmir_v22i5e16443_app2.png]

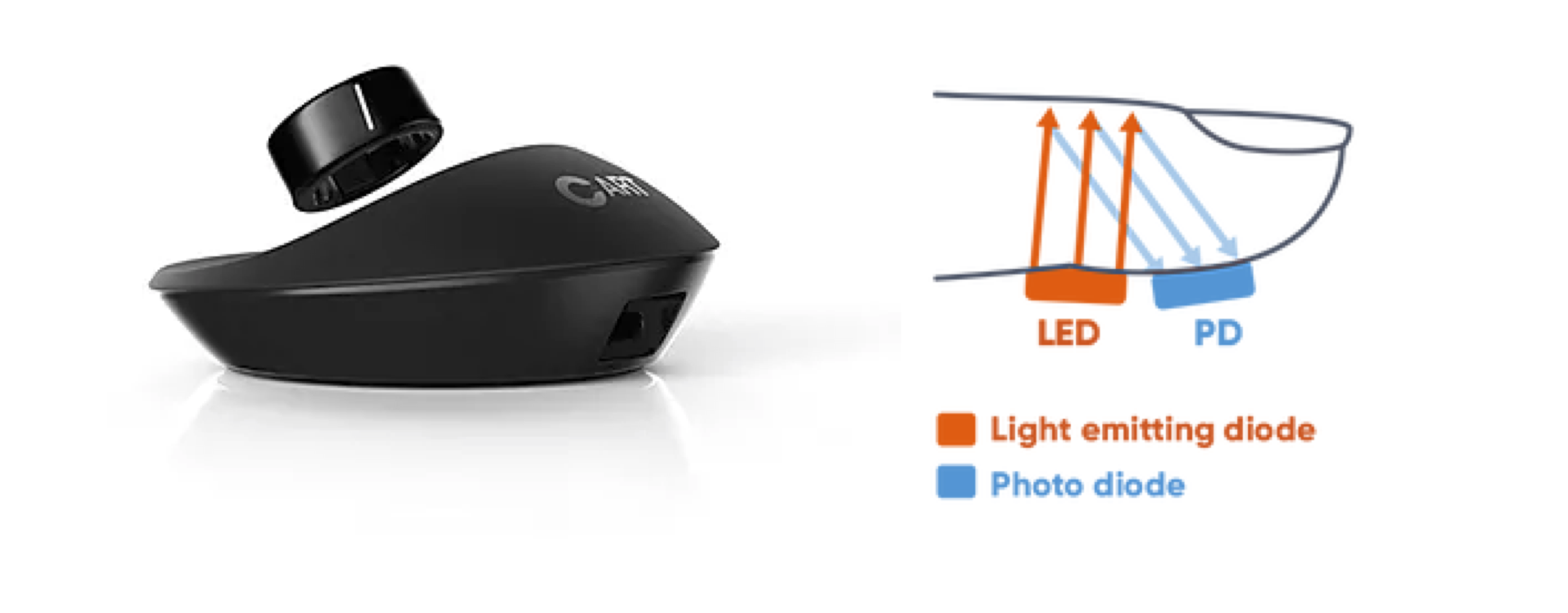

Supplement: Multimedia Appendix 3 [file jmir_v22i5e16443_app3.png]

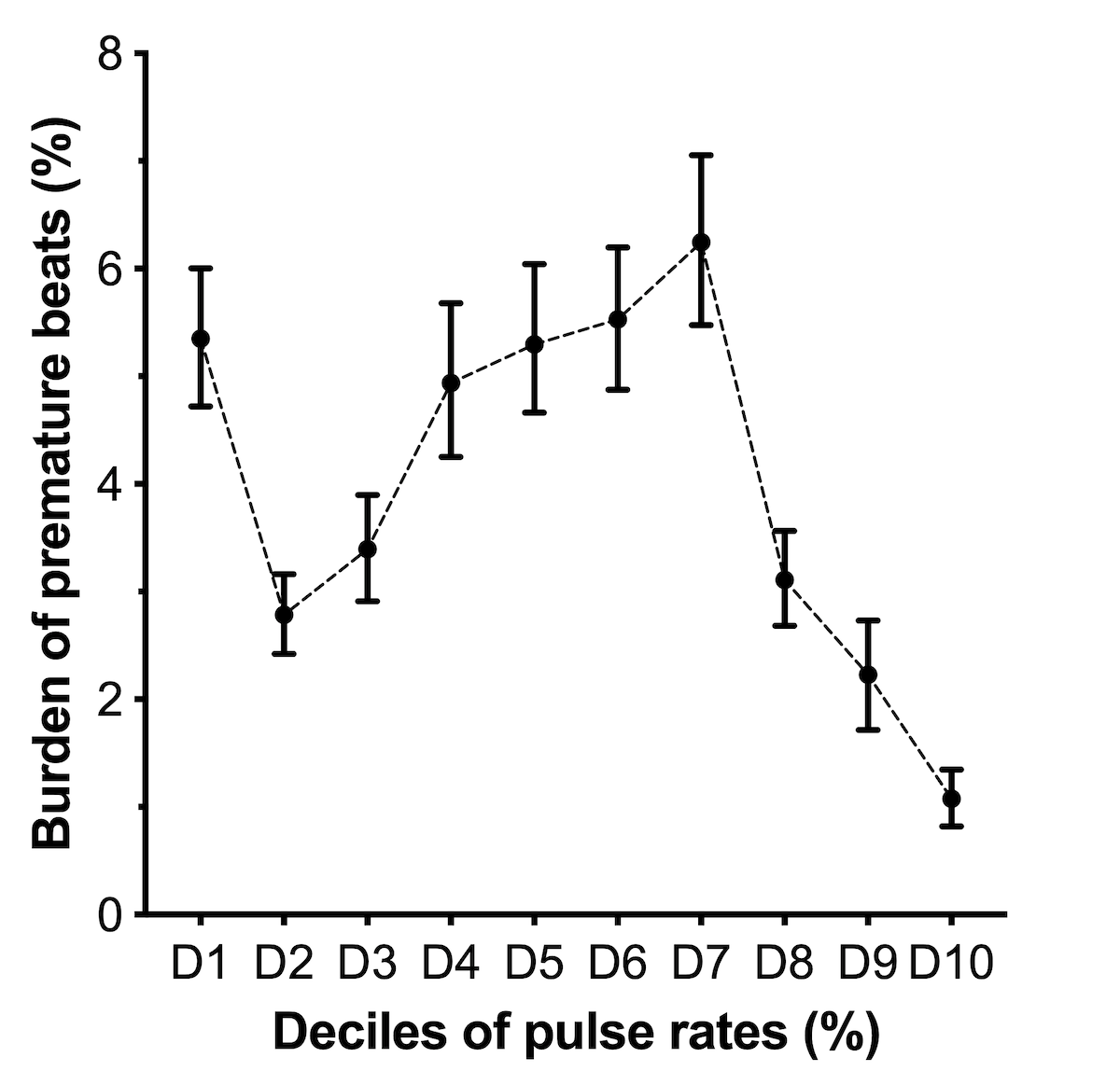

Supplement: Multimedia Appendix 4 [file jmir_v22i5e16443_app4.png]

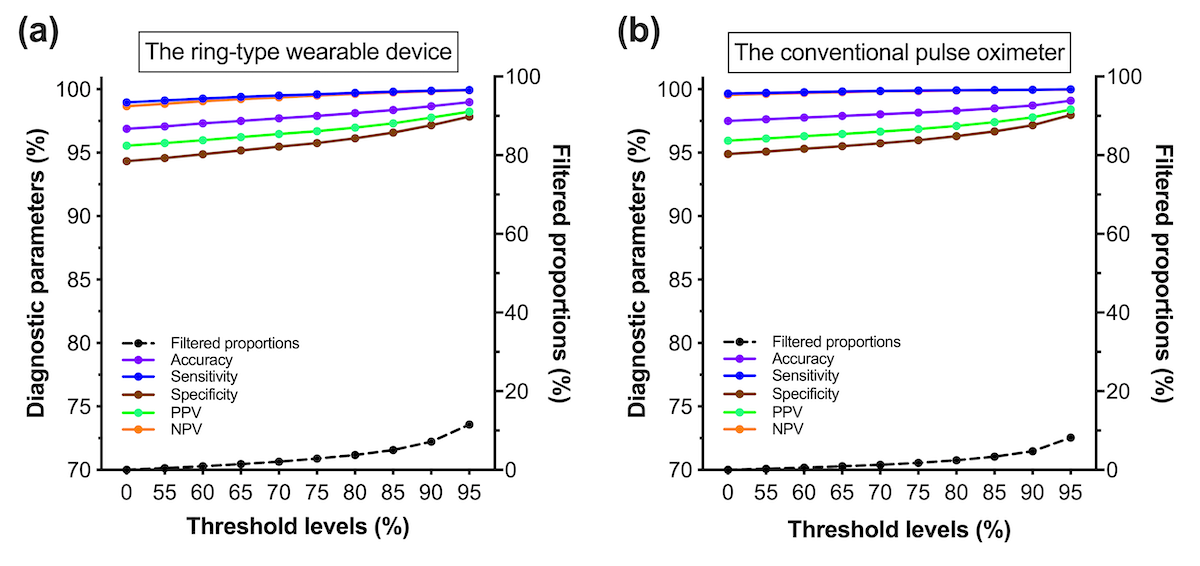

Supplement: Multimedia Appendix 5 [file jmir_v22i5e16443_app5.png]
